# Association Between Impaired Fasting Glucose and Adverse Outcomes in Patients Treated With Peritoneal Dialysis: A Retrospective Study From Southern China

**DOI:** 10.1002/edm2.70216

**Published:** 2026-05-25

**Authors:** Qinghua Hu, Qimei Luo, Jun Ai, Jiayi Chen, Jianyi Pan, Jinzhong Chen, Jun Zhang, Xianrui Dou

**Affiliations:** ^1^ Department of Nephrology, the Eighth Affiliated Hospital Southern Medical University (The First People's Hospital of Shunde, Foshan) Foshan China; ^2^ Department of Nephrology, Nanfang Hospital Southern Medical University Guangzhou China; ^3^ Department of Nephrology The Third Affiliated Hospital of Sun Yat‐Sen University Guangzhou China

**Keywords:** cardiovascular mortality, diabetes mellitus, end‐stage renal disease, impaired fasting glucose, peritoneal dialysis

## Abstract

**Aim:**

The aim of this research was to explore the relationship between impaired fasting glucose (IFG) and adverse clinical outcomes in a southern Chinese population receiving peritoneal dialysis (PD) therapy.

**Methods:**

Participants who underwent PD therapy for > 3 months during January 1, 2004 to July 27, 2021, across four centers were included. Baseline data were collected in the initial 3 months of PD therapy. We defined IFG using World Health Organization criteria (IFG‐WHO: 6.1–6.9 mmol/L). The adverse outcomes included all‐cause mortality, cardiovascular mortality and cardiovascular events. Kaplan–Meier survival analyses and Cox regression models were utilized to assess the associations between IFG and adverse outcomes.

**Results:**

A total of 2345 PD patients were included, with 666 (28.4%) diagnosed with diabetes and 209 (8.9%) with IFG. Over an average follow‐up duration of 32 months (25th–75th, 15–54), 276 (11.8%) deaths occurred, including 186 (7.9%) cardiovascular mortality, and 356 (15.2%) cardiovascular events were documented. Kaplan–Meier survival curves showed that patients with IFG had a significantly higher risk of cardiovascular mortality than those with normoglycemia. Cox multivariate analysis showed that IFG was an independent risk factor for cardiovascular mortality (HR = 1.735; 95% CI: 1.023–2.944) after adjusting for confounders. No significant differences were observed in all‐cause mortality or cardiovascular event risks between groups.

**Conclusions:**

IFG was independently associated with increased cardiovascular mortality in PD patients. These findings highlight the need for glycemic monitoring in non‐diabetic PD populations.

## Graphical abstract

This multicenter study conducted in South China showed that impaired fasting glucose was independently associated with increased cardiovascular mortality in patients treated with peritoneal dialysis. These findings highlight the need for glycemic monitoring in non‐diabetic PD populations.

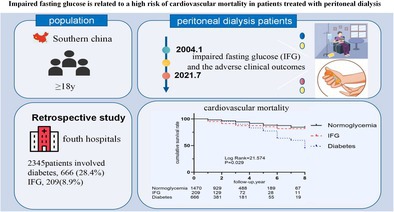

## 1 Introduction

The prevalence of end‐stage renal disease (ESRD) is increasing worldwide. Peritoneal dialysis (PD) is an extensively used renal replacement treatment for individuals with ESRD due to its advantages in maintaining hemodynamic stability and better preservation of residual renal function [1]. Glucose‐based dialysate remains the most widely used dialysis solution. Chronic exposure to large amounts of glucose from the PD fluid and the accumulation of uremic toxins such as urea and creatinine can lead to glucose metabolism disorders and insulin resistance in PD patients [2, 3].

Impaired fasting glucose (IFG) is an intermediate stage between normoglycemia and diabetes, closely associated with insulin resistance [4, 5]. It is reported that the cumulative incidence of diabetes in individuals with IFG reached 31% over 12‐year follow‐up, with a relative risk of 4.32 [6]. IFG is highly prevalent, accounting for approximately 30% of individuals in a nationally representative cross‐sectional study in the US population [7]. The diagnostic criteria for IFG vary slightly according to different guidelines. Impaired fasting glucose (IFG), defined by American Diabetes Association (ADA), as fasting plasma glucose (FPG) 5.6–6.9 mmol/L or WHO as 6.1–6.9 mmol/L [8, 9].

Previous studies have examined the associations between IFG and adverse health outcomes, revealing that compared with normoglycemia, individuals with IFG had a significantly increased risk of all‐cause mortality and elevated incidence of cardiovascular events, chronic kidney disease (CKD), and all‐cause dementia in the general population [10, 11, 12, 13, 14]. However, inconsistent conclusions exist regarding the association between IFG and mortality among PD patients. Kuan‐Hsing et al. [15] reported that baseline IFG was independently associated with the incidence of all‐cause mortality in patients on PD without diabetes, whereas a prior study found no significant association [16].

The clinical significance of mildly elevated blood glucose levels in PD patients remains uncertain. This multicenter study designates cardiovascular mortality as the primary endpoint and explores its correlation with IFG in patients undergoing PD.

### 1.1 Research Design and Methods

We retrospectively analysed data from four hospitals: The Eighth Affiliated Hospital, Southern Medical University (The First People's Hospital of Shunde, Foshan), Nanfang Hospital, Southern Medical University, First People's Hospital of Foshan, and People's Hospital, Ganzhou City. The Research Ethics Committee of The Eighth Affiliated Hospital, Southern Medical University approved the study (KYLS20231013). Written informed consent was waived because the participants' identification information was blocked during data analysis.

Initially, 3307 PD patients underwent PD for > 3 months from January 1, 2004 to July 27, 2021 were prescreened in our four centers. Exclusion criteria: (1) ≤ 18 years of age; (2) refusal to join the study; (3) lost to follow‐up; (4) taking long‐term systemic corticosteroids; (5) a history of malignancy; and (6) in combination with haemodialysis. Finally, a total of 2345 participants were enrolled and followed up at 3‐month intervals until death, PD withdrawal, or 27 December 2021.

### 1.2 Data Collection

Clinical characteristics and laboratory parameters were collected during the initial 3 months of PD therapy. Participants fasted for 10 h prior to morning blood collection and avoided interference from PD solution. Various markers were measured using standard techniques. Body mass index (BMI) was calculated using the formula: weight (kg) divided by height squared (m^2^). Overweight was defined as BMI ≥ 24.0 kg/m^2^. When hypoalbuminemia was present, the following formula was used to correct the measured serum calcium level (mmol/L): total calcium + (40 − serum albumin [g/L]) × 0.02. Residual renal function (RRF) was defined as a 24‐h urine volume > 400 mL.

### 1.3 Definitions

The definition of IFG is satisfied as follows: WHO‐IFG: FPG ranges between 110 to 125 mg/dL (6.1–6.9 mmol/L) [9], ADA‐IFG: FPG ranges between 110 to 125 mg/dL (5.6–6.9 mmol/L) [8].

The definition of diabetes is satisfied as follows: (i) FPG ≥ 7.0 mmol/L, (ii) 2‐h plasma glucose ≥ 11.1 mmol/L in an oral glucose tolerance test (OGTT) or (iii) HbA1c ≥ 6.5%, (v) diabetes symptoms with random blood glucose ≥ 11.1 mmol/L, or (v) a history of physician diagnosis, or (vi) using diabetes medication [9].

### 1.4 Outcomes

The primary outcome was CVD‐specific mortality. The secondary outcomes were CVD incidence and all‐cause mortality. CVD encompasses coronary artery disease, heart failure, atrial fibrillation, any stroke, and any ischemic stroke [17]. All these endpoint events were obtained from the medical records and were determined by at least two nephrologists at the PD centers.

### 1.5 Statistical Analysis

The baseline characteristics of the subjects were summarized as means ± standard deviations or median for continuous variables, and proportions for categorical variables, respectively. Participants were categorized into three groups based on initial FPG levels per the WHO guideline criteria: (i) Normoglycemia (FPG < 6.1 mmol/L), (ii) IFG (FPG 6.1–6.9 mmol/L), (iii) Diabetes (FPG ≥ 7.0 mmol/L or prior diagnosis). Intergroup comparisons used ANOVA for normally distributed continuous variables, Kruskal–Wallis H test for non‐normally distributed data, and χ^2^ tests for categorical variables.

Survival curves were compared using the Kaplan–Meier method with log‐rank test. Cox proportional hazards models (both univariate and multivariate) assessed outcome risks among normoglycemia (< 6.1 mmol/L), impaired fasting glucose (IFG, 6.1–6.9 mmol/L), and diabetes (≥ 7.0 mmol/L) groups. Multivariate models adjusted for clinically relevant covariates: age, sex, BMI, hypertension status, 24‐h urine volume, haemoglobin, and serum albumin levels. We further stratified patients into < 5.6 mmol/L, 5.6–6.0 mmol/L and 6.1–6.9 mmol/L subgroups for additional sensitivity analyses using χ^2^ tests and Cox models.

Univariate logistic regression analysis initially identified potential risk factors for IFG in PD patients. Variables with *p* < 0.05 in univariate analysis and clinically relevant covariates including sex, overweight status, and lower serum high‐density lipoprotein (HDL) were included in the multivariable logistic regression model. All analyses of statistical data were conducted using SPSS version 27. Statistical significance was set at *p* values < 0.05.

## 2 Results

### 2.1 Baseline Characteristics

**FIGURE 1:**
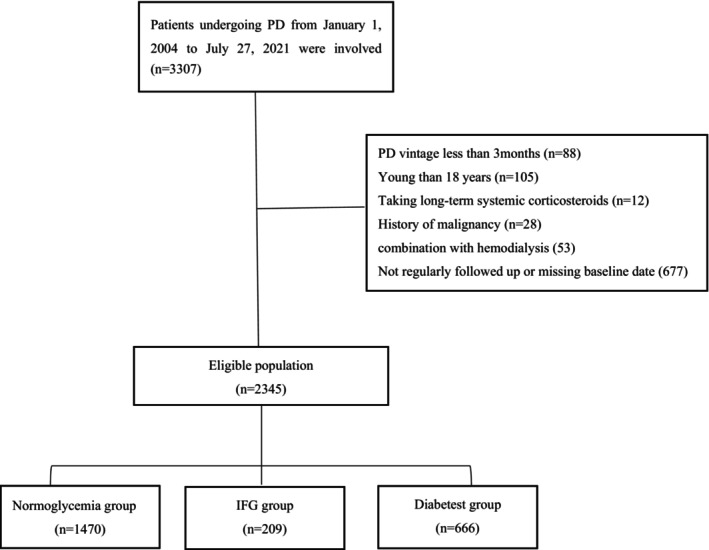
Flowchart of participant enrollment. Patients undergoing PD from January 1, 2004 to July 27, 2021 were involved (*n* = 3307).

A total of 3307 participants who underwent PD were initially screened from four centers, of which 963 were excluded. Ultimately, 2345 participants were enrolled [normoglycemia group, *n* = 1470 (62.7%); IFG group, *n* = 209 (8.9%); and diabetes group, *n* = 666 (28.4%)] (Figure 1).

The average age of participants was 49.9 ± 13.8 years, with a median duration of dialysis treatment at 32 months (25th–75th, 15–54). Among the participants, 1309 (55.8%) were male. Compared with the normoglycemia group, patients with IFG were significantly older and had a higher level of C‐reactive protein (CRP), blood urea nitrogen (BUN) levels, and hypertension prevalence, but lower lymphocyte counts (Table 1).

**TABLE 1:** Comparative analysis of baseline characteristics in PD patients: A retrospective study.

Variables	Total (*n* = 2345)	Normoglycemia (*n* = 1470)	IFG (*n* = 209)	Diabetes (*n* = 666)
Age, years	47.0 ± 14.0	44.4 ± 13.6	47.0 ± 14.4^a^	52.9 ± 12.9^a^, ^b^
Male, no. (%)	1309 (55.8)	782 (53.2)	112 (53.6)	415.0 (62.3)
BMI, kg/m^2^	21.9 ± 3.2	21.6 ± 3.2	21.4 ± 2.8	22.7 ± 3.279^a^, ^b^
Hypertension, no. (%)	2085 (89.0)	1280 (87.1)	189 (90.9)	616 (92.5)
Smoking, no. (%)	354 (15.1)	205 (13.9)	26 (12.4)	123 (18.5)
Drinking, no. (%)	208 (8.9)	129 (8.8)	10 (4.8)	69 (10.4)
PD vintage, months	32 (15, 55)	33 (16, 55)	34 (15, 56)	28 (12, 49)9^a^, ^b^
SBP, mmHg	142.4 ± 21.1	142.0 ± 21.0	140.9 ± 18.7	143.7 ± 21.7
DBP, mmHg	86.2 ± 13.0	87.6 ± 12.8	86.6 ± 13.0	83.2 ± 12.99^a^, ^b^
24‐h urine volume, ml/24 h	1000.0 (600.0,1200.0)	1120.0 (900.0,1500.0)	1100.0 (525.0,1725.0)	1400.0 (1000.0,1800.0)
WBC count, 10^9^/L	7.0 ± 3.2	6.8 ± 2.4	6.9 ± 2.5	7.4 ± 4.59^a^, ^b^
Lymphocyte, 10^9/L	11.3 ± 11.3	13.3 ± 11.7	6.2 ± 9.3^a^	8.5 ± 9.99^a^, ^b^
Haemoglobin, g/L	83.3 ± 19.5	83.6 ± 19.4	80.9 ± 20.0	83.6 ± 19.6
Phosphorus, mmol/L	4.6 (4.0, 5.1)	4.5 (4, 5.28)	4.85 (4.4, 5.3)	4.6 (4.1, 5.1)
Calcium, mmol/L	2.1 (1.9, 2.3)	2.1 (2.0, 2.2)	2.2 (1.9, 2.3)	2.2 (2.1, 2.3)
Potassium, mmol/L	2.0 (1.6, 2.4)	2.1 (1.7, 2.6)	2.6 (2.1, 2.9)	1.9 (1.5, 2.3)
BUN, mmol/L	28.9 ± 12.3	28.2 ± 12.0	30.5 ± 12.6^a^	29.8 ± 13.0^a^
Creatinine, μmol/L	973.8 ± 368.9	989.2 ± 375.6	1011.8 ± 334.7	927.9 ± 360.59^a^, ^b^
Uric acid, μmol/L	513.0 ± 155.1	517.7 ± 157.4	521.2 ± 153.3	499.8 ± 149.9^a^
Albumin, g/L	36.3 ± 5.4	36.5 ± 5.3	37.0 ± 5.2	35.7 ± 5.69^a^, ^b^
TG, mmol/L	1.3 (1.0, 1.9)	1.3 (0.9, 2.0)	1.4 (0.9, 2.1)	1.2 (0.9, 1.9)
HDL, mmol/L	1.1 (0.9, 1.4)	1.1 (0.9, 1.5)	1.0 (0.9, 1.4)	1.1 (0.9, 1.5)
LDL, mmol/L	2.5 (2.0, 3.1)	2.9 (2.2, 3.2)	2.7 (2.0, 3.2)	2.7 (1.9, 3.2)
CRP, mg/L	4.6 (1.9, 10.6)	3.6 (1.6, 8.9)	5.0 (3.1, 11.0)^a^	5.0 (3.0, 14.2)^a^
iPTH, pg/ml	294.7 (136, 489.4)	60.4 (27.0, 278.1)	53.6 (45.0, 217.4)	52.0 (27, 7192.8)^a^
Serumiron, μmol/L	10.0 (7.0, 14.0)	10.7 (7.5, 13.3)	9.4 (6.3, 11.2)	10.7 (7.5, 16.2)
Ferritin, ug/L	223.8 (91, 3428.9)	203.3 (57, 2410.1)	207.4 (71, 5470.3)	226.2 (97, 3422.2)

*Note:* Continuous variables are presented as mean ± SD or median (25th percentile‐75th percentile), category variables are presented as *n* (%). Normoglycemia, FPG < 6.1 mmol/L; IFG, FPG within 6.1–6.9 mmol/L; Diabetes, FPG ≥ 7.0 mmol/L or prior diagnosis.

Abbreviations: BMI, body mass index; BUN, blood urea nitrogen; CRP, C‐reactive protein; DBP, diastolic blood pressure; HDL, high density lipoprotein; LDL, low density lipoprotein; SBP, systolic blood pressure; TG, triglyceride; WBC count, white blood cells.

^a^ IFG and diabetes versus Normoglycemia, *p* < 0.05.

^b^ IFG versus diabetes, *p* < 0.05.

### 2.2 Association Between IFG and Adverse Outcomes in PD Patients

**FIGURE 2:**
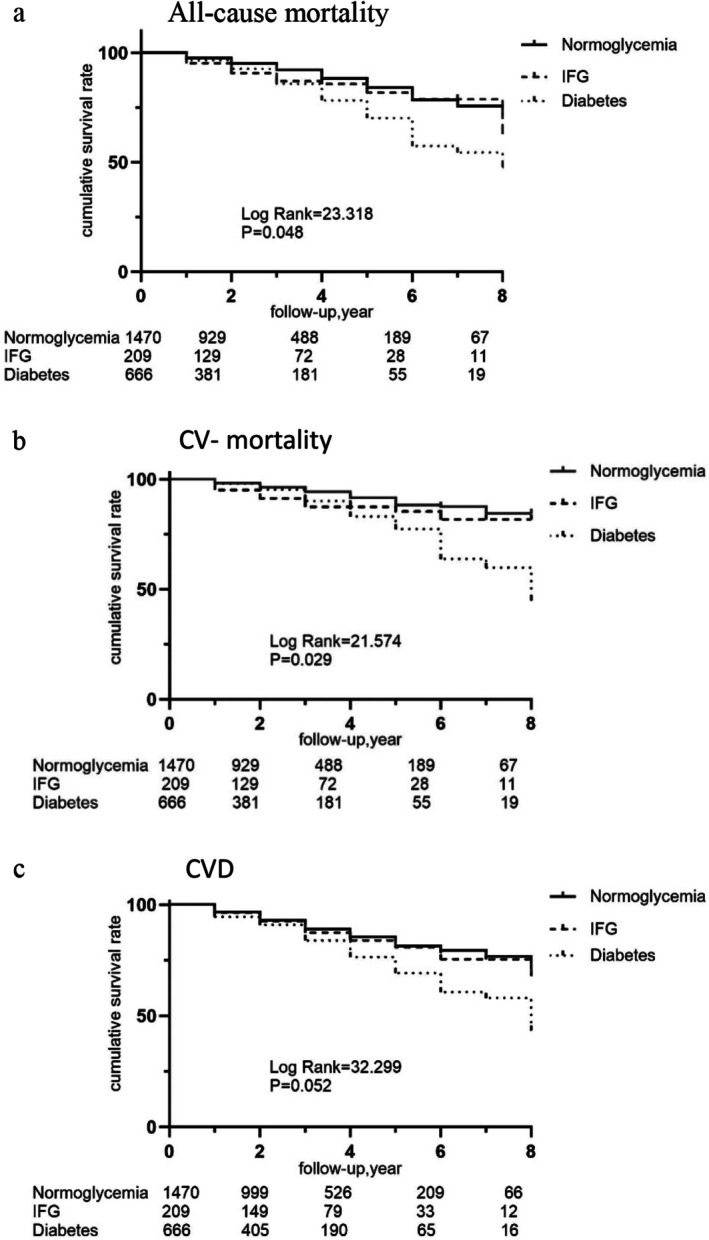
Kaplan–Meier survival analyses of clinical outcomes in PD patients. (a) All‐cause mortality in PD patients (Normoglycemia vs. IFG *p* = 0.117, Normoglycemia vs. diabetes *p* < 0.001, IFG vs. diabetes *p* = 0.17). (b) CV‐mortality in PD patients (Normoglycemia vs. IFG *p* = 0.02, Normoglycemia vs. diabetes *p* < 0.001, IFG vs. diabetes *p* = 0.456). (c) CVD in PD patients (Normoglycemia vs. IFG *p* = 0.444, Normoglycemia vs. diabetes *p* < 0.001, IFG vs. diabetes *p* = 0.0110). Normoglycemia, FPG < 6.1 mmol/L; IFG, FPG within 6.1–6.9 mmol/L; Diabetes, FPG ≥ 7.0 mmol/L or prior diagnosis. CVD, cardiovascular disease; CV‐mortality, cardiovascular mortality; FPG, fasting plasma glucose; FG, impaired fasting glucose.

Over a median follow‐up of 32 months (25th–75th, 15–54), 276 patients (11.8%) died, including 186 (7.9%) cardiovascular deaths, while 356 (15.2%) developed composite CVD (Table 2). Kaplan–Meier analysis demonstrated higher cardiovascular mortality in the IFG group versus normoglycemia (*p* = 0.02), but no significant differences in all‐cause mortality or CVD incidence (Figure 2).

**TABLE 2:** Cox regression analysis for FPG stratification with clinical outcomes in PD patients.

Group	No. Events	Unadjusted		Adjusted	
		HR (95% CI)	*p*	HR (95% CI)	*p*
All‐cause mortality	276 (11.8%)				
Normoglycemia	145 (9.9%)	0	0	0	0
IFG	28 (13.4%)	1.36 (0.9, 2.04)	0.137	1.374 (0.87, 2.159)	0.168
Diabetes	103 (15.5%)	1.88 (1.46, 2.42)	0.01	1.399 (1.040, 1.881)	0.026
CV‐mortality	186 (7.9%)				
Normoglycemia	92 (6.3%)	0	0	0	0
IFG	22 (10.5%)	1.71 (1.07, 2.72)	0.025	1.455 (1.018, 2.080)	0.039
Diabetes	72 (10.8%)	2.07 (1.52, 2.82)	0.01	1.708 (1.029, 2.836)	0.038
CVD	356 (15.2%)				
Normoglycemia	189 (12.9%)	0	0	0	0
IFG	31 (14.8%)	1.157 (0.796, 1.683)	0.445	1.127 (0.748, 1.699)	0.566
Diabetes	136 (20.4%)	1.866 (1.497, 2.327)	0.01	1.632 (1.275, 2.088)	0.01

*Note:* Adjusted group: adjust for age, sex, BMI, hypertension status, 24‐h urine volume, albumin and haemoglobin. Normoglycemia, FPG < 6.1 mmol/L; IFG, FPG within 6.1–6.9 mmol/L; Diabetes (FPG ≥ 7.0 mmol/L or prior diagnosis).

Abbreviations: CV‐mortality, cardiovascular mortality; CVD, cardiovascular disease; IFG, impaired fasting glucose.

The relationship between initial FPG and the associated outcomes was evaluated using multivariate Cox proportional hazards regression analysis. As shown in Table 2, compared with the reference group, the IFG group in the unadjusted model (Model 1) had significantly elevated risks of cardiovascular mortality [HR = 1.71; 95% CI: 1.07–2.72], with no significant associations with all‐cause mortality or CVD. After adjusting for variables such as age, sex and hypertension status, BMI, 24‐h urine volume, haemoglobin and albumin (Model 2), baseline IFG remained an independent risk factor for cardiovascular mortality [HR = 1.455; 95% CI: 1.018–2.080]. All three outcomes showed significantly increased risks among individuals with diabetes versus normoglycemia (all *p* < 0.05) (Table 2).

We further performed a stratified analysis; none of the variables including smoking (no vs. yes), age (< 60 vs. ≥ 60 years), hypertension status (no vs. yes), presence of RRF (no vs. yes), albumin (< 40 vs. ≥ 40 g/L), and BMI (< 24 vs. ≥ 24 kg/m2) considerably modified the association of IFG and risk of cardiovascular mortality in patients on PD (Figure S1).

### 2.3 Heterogeneity in IFG Diagnostic Criteria and Adverse Outcomes Among PD Patients

Table S1 presents the demographic characteristics of participants with IFG defined by ADA criteria (5.6–6.9 mmol/L). Multivariable Cox proportional hazard analyses demonstrated no significant differences in CVD incidence or mortality between ADA‐defined IFG and normoglycemia groups (Table S1).

**FIGURE 3:**
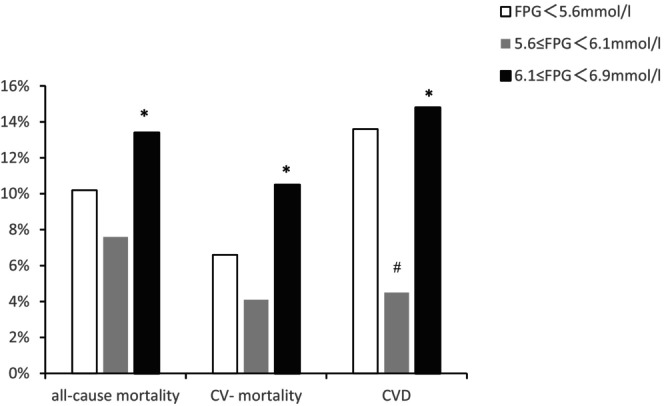
Incidence of clinical outcomes by FPG stratification in PD patients. CVD, cardiovascular disease; CV‐mortality, cardiovascular mortality; FPG, fasting plasma glucose. (^#^FPG < 5.6 mmol/L vs. 5.6 ≤ FPG < 6.1 mmol/L, *p* < 0.05; ^▲^FPG < 5.6 mmol/L vs. 6.1 ≤ FPG < 6.9 mmol/L, *p* < 0.05; *5.6 ≤ FPG < 6.1 mmol/L vs. 6.1 ≤ FPG < 6.9 mmol/L, *p* < 0.05).

To assess the dose–response relationship, non‐diabetic PD patients were divided into three groups (< 5.6 mmol/L, 5.6–6.0 mmol/L, 6.1–6.9 mmol/L) according to FPG. As showed in Figure 3, compared with the < 5.6 mmol/L and 6.1–6.9 mmol/L groups, PD patients in the 5.6–6.1 mmol/L group demonstrated the lowest incidence of adverse outcomes. No significant difference in adverse outcomes was observed between PD patients with FPG < 5.6 mmol/L and those with 6.1 ≤ FPG < 6.9 mmol/L (Figure 3). Further Cox regression analysis revealed that compared with 5.6–6.0 mmol/L group, the < 5.6 mmol/L group showed significantly higher composite CVD risk (HR = 1.771; 95% CI: 1.021–3.072), the 6.1–6.9 mmol/L group had 135% higher all‐cause mortality (HR = 2.35; 95% CI: 1.066–5.181), 169% higher cardiovascular mortality (HR = 2.687; 95% CI: 1.054–6.848) (Tables S1 and S2).

### 2.4 Risk Factors for IFG in Patients on PD

Univariate logistic regression analysis initially screened potential predictors of IFG in PD patients. Covariates with *p* < 0.05 on univariate analysis such as age, BUN, TG and clinically relevant covariates as shown in Table 3 were subsumed into the multivariable logistic regression model. The final adjusted model identified these independent risk factors for IFG: age ≥ 60 years (OR = 1.934, 95% CI 1.239–3.018), TG ≥ 2.22 mmol/L (OR = 1.671, 95% CI 1.008–2.770), elevated blood urea nitrogen (per 1 mmol/L increase: OR = 1.025, 95% CI 1.010–1.041) (Table 3).

**TABLE 3:** Risk factors for IFG in PD patients.

Variable	Unadjusted OR (95% CI)	*p*	Adjusted OR (95% CI)	*p*
Sex	0.984 (0.736, 1.316)	0.916	1.119 (0.757, 1.656)	0.572
Smoking	0.877 (0.567, 1.357)	0.555		
Age, years (< 60, ≥ 60)	1.502 (1.055, 2.138)	0.024	1.934 (1.239, 3.018)	0.01
BMI, kg/m^2^	0.982 (0.936, 1.03)	0.449	0.986 (0.926, 1.050)	0.654
Hypertension status (0 = no, 1 = yes)	1.477 (0.899, 2.424)	0.123	0.863 (0.489, 1.521)	0.61
BUN, mmol/L	1.014 (1.003, 1.026)	0.013	1.025 (1.01, 1.041)	0.001
Creatinine, μmol/L	1 (1, 1.001)	0.409		
Urine volume, ml/24 h	1.157 (0.691, 1.935)	0.579		
Haemoglobin, g/L	0.996 (0.988, 1.003)	0.254		
TG, mmol/L (< 2.22, ≥ 2.22)	1.634 (1.119, 2.386)	0.011	1.671 (1.008, 2.77)	0.046
HDL, mmol/L (< 0.9, ≥ 0.9)	1.285 (0.779, 2.12)	0.327	1.469 (0.863, 2.201)	0.156

*Note:* IFG, FPG within 6.1–6.9 mmol/L.

Abbreviations: BMI, body mass index; BUN, blood urea nitrogen; HDL, high density lipoprotein; TG, triglyceride.

## 3 Discussion

Our multicenter study reveals three principal observations. First, IFG (WHO‐defined: 6.1–6.9 mmol/L) independently increases cardiovascular mortality risk in PD patients (HR = 1.735, 95% CI: 1.02–2.94), whereas ADA‐defined IFG (5.6–6.9 mmol/L) showed inferior predictive value. Second, the WHO criteria better discriminated high‐risk PD patients, likely due to exclusion of borderline elevations (5.6–6.0 mmol/L) with lower clinical significance. Third, we identified three modifiable risk factors for IFG in PD patients: age ≥ 60 years, hypertriglyceridemia (≥ 2.22 mmol/L), and higher BUN.

FPG demonstrates superior clinical utility in PD populations, owing to its lower susceptibility to dialysate glucose interference and higher feasibility than OGTT or HbA1c measurements [18, 19]. Its variability in uremia necessitates cautious interpretation. Elevated FPG consistently predicted mortality across studies [20, 21], supporting its use despite physiological perturbations.

Our present multicenter study showed that, even in the complex internal environment of uremia, baseline IFG was still associated with an elevated cardiovascular mortality risk in patients who underwent PD treatment. The comparable cardiovascular mortality between IFG (10.3%) and diabetic (10.8%) groups may reflect shared insulin resistance pathways [22, 23]. PD‐specific factors including chronic glucose loading from dialysate likely exacerbate atherosclerosis through endothelial dysfunction [24, 25]. This dual metabolic burden underscores the need for tailored screening in PD populations.

Current evidence regarding IFG and clinical outcomes in PD patients demonstrates significant heterogeneity across studies; these discrepancies likely stem from variations in diagnostic thresholds (WHO vs. ADA criteria), population characteristics, and insufficient statistical power in most cohorts [16, 26, 27, 28]. Despite IFG's high prevalence and substantial clinical implications, no consensus exists on its diagnostic criteria.

Some prospective studies based on the general population have shown that the presence of ADA‐IFG was associated with an increased risk of cardiovascular disease, whereas others have obtained inconsistent results [29, 30, 31]. A recent meta‐analysis [32] of > 10 million participants clarified this controversy, demonstrating that cardiovascular mortality risk was predominantly associated with the upper IFG range (6.1–6.9 mmol/L), aligning with WHO criteria. The optimal diagnostic criteria for IFG in PD patients remain uncertain.

Szeto et al. [27] established that even mild hyperglycemia (FPG > 5.6 mmol/L) significantly reduces survival in PD patients. In contrast, Yiping et al. [16] demonstrated no statistically significant differences in all‐cause mortality across FPG strata (< 5.6 mmol/L and 5.6–6.9 mmol/L) in their adjusted Cox regression analysis. Our multicenter study of Southern Chinese PD patients further clarified this dichotomy: while WHO‐defined IFG (FPG 6.1–6.9 mmol/L) independently predicted cardiovascular mortality (HR = 1.73, 1.02–2.94), ADA‐defined thresholds (5.6–6.9 mmol/L) showed no significant association. This discrepancy reflected the limited prognostic value of ADA‐defined IFG thresholds in PD populations.

To quantify the dose–response relationship in PD patients, participants were categorized into three groups based on FPG levels: the < 5.6 mmol/L group, the 5.6–6.0 mmol/L group, and the 6.1–6.9 mmol/L group. Multivariable Cox regression demonstrated the reference range (5.6–6.0 mmol/L) optimal prognosis: 6.1–6.9 mmol/L versus 5.6–6.0 mmol/L conferred 135% higher all‐cause mortality (HR = 2.35; 95% CI: 1.066–5.181) and 169% increased cardiovascular mortality (HR = 2.687; 95% CI: 1.054–6.848). FPG < 5.6 mmol/L versus 5.6–6.0 mmol/L elevated composite CVD risk by 77.1% (HR = 1.771; 95% CI: 1.021–3.072). Accumulating evidence confirms a J‐shaped relationship between FPG levels and CVD risk in dialysis populations [33, 34, 35]. The elevated risk at both glycemic extremes may reflect distinct pathophysiological mechanisms: the higher glucose range (6.1–6.9 mmol/L) promotes atherosclerotic progression through advanced glycation end‐products, whereas the lower glucose range (< 5.6 mmol/L) signifies malnutrition‐inflammation complex syndrome, a well‐established driver of cardiovascular mortality in ESRD. Our study suggests that a targeted FPG range of 5.6–6.1 mmol/L may represent the optimal glycemic control window for PD patients, underscoring the need for dialysis‐specific glucose management criteria. Large‐scale cohort studies involving different ethnic groups may be required in future to establish specialized criteria for IFG in PD patients.

Older age, dyslipidemia, obesity and genetic predisposition are established risk factors for IFG in the general population [10]. Our study presented similar associations in PD patients, identifying age ≥ 60 years and hypertriglyceridemia (≥ 2.22 mmol/L) as independent predictors. It is remarkable that BUN was an independent risk factor for IFG in this population. While diabetes mellitus unquestionably accelerates kidney disease progression, the latest evidence indicates a bidirectional relevance between these conditions [36, 37]. Koppe et al. [38] and Thomas et al. [23] have suggested that elevated levels of circulating urea directly impair insulin sensitivity and β‐cell secretory function in the advanced stages of CKD through experimental evidence. Xie Y et al. [36] studied a cohort of 1,337,452 US veterans without diabetes and demonstrated that an elevated BUN level was relevant to an increased risk of incident diabetes mellitus in advanced kidney disease. The risk of diabetes was higher in those with a higher BUN level (> 25 mg/dL) irrespective of estimated glomerular filtration rate (eGFR), and the relevance between eGFR and the risk of diabetes was not significant in those with a BUN level < 25 mg/dL. Our study provides the first evidence that BUN concentration independently predicts IFG risk in PD patients. Quantitatively, each 1 mmol/L increase in BUN level correlated with a 3.8% absolute rise in IFG incidence. Further studies are necessary to validate these findings in the future through larger cohorts, to examine whether interventions (pharmacological means and low‐protein diet) to reduce urea or its downstream effects will lead to a reduction in the risk of diabetes in patients on PD.

Although usually asymptomatic, IFG represents a critical opportunity to restrict progression to T2DM and its complications [39, 40]. Our results provide evidence that IFG should be emphasized for PD patients, especially prioritizing screening in high‐risk patients with advanced age (≥ 60 years) and metabolic syndrome components (BMI ≥ 24 kg/m^2^ or triglycerides ≥ 2.2 mmol/L). For PD patients diagnosed with IFG, an intensive glycemic monitoring protocol is needed in order to detect T2DM and its complications in time. Although amino acid‐based dialysate and icodextrin can mitigate the glycemic impact of glucose‐based dialysate, their high cost (approximately 2–3 times that of conventional glucose‐based dialysate) limits their widespread clinical application, especially in low and middle‐income regions [41, 42]. It is worthy to research whether intervention measures, including medication or lifestyle modifications such as exercise training and weight loss, prevent the occurrence of diabetes in PD patients.

## 4 Limitations

Our study has several strengths. First, by contrasting WHO (6.1–6.9 mmol/L) and ADA (5.6–6.9 mmol/L) IFG thresholds, we established that only WHO‐defined IFG independently predicted cardiovascular mortality, addressing a critical diagnostic dilemma in PD populations. Subsequently, through further evaluating the dose–response relationship between blood glucose levels and prognostic risk in PD patients, we identified that the 5.6–6.0 mmol/L showed optimal prognosis compared with < 5.6 mmol/L and 6.1–6.9 mmol/L. These differentials reflected the limited prognostic value of ADA‐defined IFG thresholds in this high‐risk population. Second, this was a multicenter study; the four hospitals from which the patients were recruited were representative of the southern region of China. Third, all baseline data were collected during hospitalization to ensure that patients undergoing blood glucose measurement fasted for 10 h and avoid affected by the PD solution on the sample. Fourth, all endpoint data were strictly assessed using medicines to ensure data accuracy.

This study had several limitations. First, IFG was classified based on a solitary measurement at baseline. While this method is frequently employed in previous research, it might lead to erroneous classification for certain individuals. Second, although this was conducted as a multicenter study, the populations were geographically restricted to Southern China, potentially limiting generalizability to populations with distinct genetic backgrounds and dietary patterns. Future validation through real‐world studies with larger sample sizes across diverse populations is warranted. Third, given the median follow‐up duration of 32 months (25th–75th, 15–54), extended longitudinal observation is required to comprehensively assess the occurrence of late‐onset complications in this patient population.

## 5 Conclusion

Impaired fasting glucose (IFG) was independently associated with increased cardiovascular mortality in PD patients, though its association with all‐cause mortality and cardiovascular events did not reach statistical significance. These findings highlight the need for glycemic monitoring in non‐diabetic PD populations.

## Author Contributions

**Jinzhong Chen:** conceptualization, methodology, software, data curation, project administration, visualization. **Jiayi Chen:** data curation, resources, project administration, writing – review and editing. **Xianrui Dou:** funding acquisition, investigation, conceptualization, methodology, validation, visualization, project administration, supervision, resources, writing – review and editing, writing – original draft. **Qinghua Hu:** methodology, writing – review and editing, writing – original draft, funding acquisition, project administration, data curation, validation, investigation, conceptualization. **Jun Zhang:** resources, project administration, visualization, supervision, formal analysis, validation, investigation. **Jianyi Pan:** data curation, formal analysis, project administration, investigation.

## Funding

This study was supported by the Science and Technology Project of Foshan (Grant No. 2320001007513). The funders had no role in the design and conduct of the study; collection, management, analysis, and interpretation of the data; preparation, review, or approval of the manuscript; and decision to submit the manuscript for publication.

## Conflicts of Interest

The authors declare no conflicts of interest.

## Supporting information


**Figure S1:** Impact of IFG on cardiovascular mortality in PD Patients: a subgroup‐stratified analysis.
**Table S1:** Cox regression analysis for the association between different FPG and outcomes in PD patients.
**Table S2:** Cox regression analysis for the association between different FPG and outcomes in PD patients.

## Data Availability

The data that support the findings of this study are openly available in figshare at https://doi.org/10.6084/m9.figshare.30032437.v1, reference number doi:10.6084/m9.figshare.30032437.
