# Supplementary material for: Association Between Impaired Fasting Glucose and Adverse Outcomes in Patients Treated With Peritoneal Dialysis: A Retrospective Study From Southern China
Source: Endocrinol Diabetes Metab. 2026 May 25;9(3):e70216. doi: 10.1002/edm2.70216 (PMC13240550; doi:10.1002/edm2.70216)
Supplement: Supplementary file 1 — Figure S1: Impact of IFG on cardiovascular mortality in PD Patients: a subgroup‐stratified analysis. Table S1: Cox regression analysis for the association between different FPG and outcomes in PD patients. Table S2: Cox regression analysis for the association between different FPG and outcomes in PD patients. [file EDM2-9-e70216-s001.docx]

**Supplementary Figure 1. Impact of IFG on cardiovascular mortality in PD Patients: a subgroup-stratified analysis.**
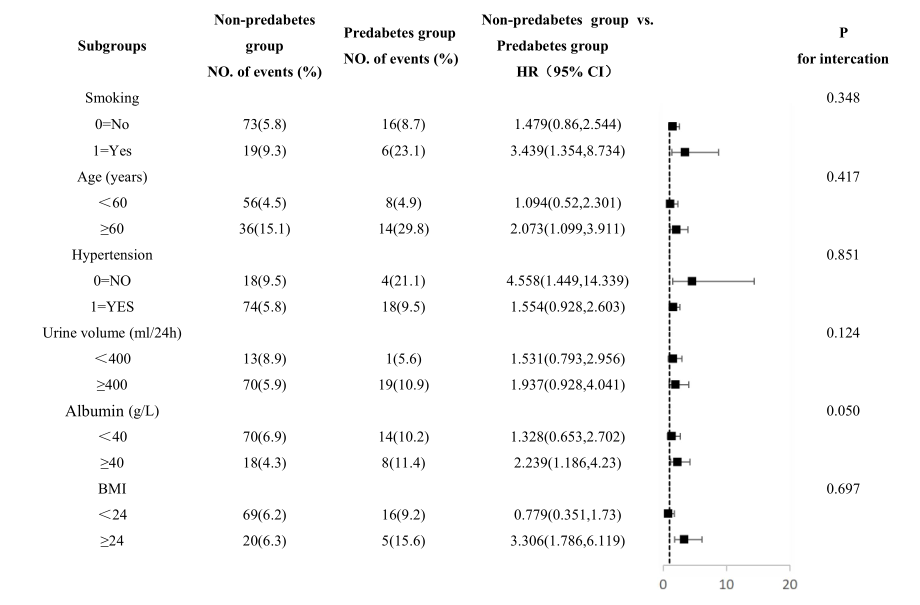


Footnotes: IFG, FPG within 6.1–6.9 mmol/L.

Abbreviations: BMI, body mass index.

**Supplementary Tabel1. Cox regression analysis for the association between different FPG and outcomes in PD patients.**

| Group | No. Events | Unadjusted | | Adjusted | |
| --- | --- | --- | --- | --- | --- |
|  |  | HR (95% CI) | P value | HR (95% CI) | P value |
| All-cause mortality | 276(11.8%) |  |  |  |  |
| FPG<5.6mmol/L | 130（10.2%） | 0 | 0 | 0 | 0 |
| 5.6≤FPG≤6.9mmol/L | 42（10.4%） | 0.905(0.638,1.284） | 0.577 | 0.917(0.612,1.374） | 0.673 |
| 6.1≤FPG<6.9mmol/L | 28（13.4%） | 1.28(0.85,1.926） | 0.237 | 1.294(0.820,2.041） | 0.268 |
| CV-mortality | 186(7.9%) |  |  |  |  |
| FPG<5.6mmol/L | 84（6.6%） | 0 | 0 | 0 | 0 |
| 5.6≤FPG≤6.9mmol/L | 29（7.2%） | 0.970(0.634,1.483） | 0.887 | 1.070(0.671,1.706） | 0.777 |
| 6.1≤FPG<6.9mmol/L | 22（10.5%） | 1.573(0.982,2.517） | 0.059 | 1.613(0.967,2.691） | 0.067 |
| CVD | 356(15.2%) |  |  |  |  |
| FPG<5.6mmol/L | 173（13.6%） | 0 | 0 | 0 | 0 |
| 5.6≤FPG≤6.9mmol/L | 46（11.4%） | 0.732(0.528,1.015） | 0.062 | 0.773(0.542,1.103） | 0.156 |
| 6.1≤FPG<6.9mmol/L | 31（14.8%） | 1.054(0.718,1.545） | 0.789 | 1.020(0.670,1.552） | 0.928 |

Adjusted group: adjusted for age, gender, BMI, hypertension status, 24-h urine volume, albumin, hemoglobin.

Abbreviations: CV-mortality, cardiovascular mortality; CVD, cardiovascular disease; FPG: fasting plasma glucose.

**Supplementary Tabel2. Cox regression analysis for the association between different FPG and outcomes in PD patients.**

| Group | No. Events | Unadjusted | | Adjusted | |
| --- | --- | --- | --- | --- | --- |
|  |  | HR (95% CI) | P value | HR (95% CI) | P value |
| All-cause mortality | 276(11.8%) |  |  |  |  |
| 5.6≤FPG<6.1mmol/L | 15（7.6%） | 0 | 0 | 0 | 0 |
| FPG<5.6mmol/L | 130（10.2%） | 1.645(0.957,2.828） | 0.072 | 1.809(0.910,3.596） | 0.091 |
| 6.1≤FPG<6.9mmol/L | 28（13.4%） | 2.085(1.093,3.976） | 0.026 | 2.350(1.066,5.181） | 0.034 |
| CV-mortality | 186(7.9%) |  |  |  |  |
| 5.6≤FPG<6.1mmol/L | 8（4.1%） | 0 | 0 | 0 | 0 |
| FPG<5.6mmol/L | 84（6.6%） | 2.025(0.966,4.246） | 0.062 | 1.809(0.781,4.194） | 0.167 |
| 6.1≤FPG<6.9mmol/L | 22（10.5%） | 3.252(1.382,7.652） | 0.007 | 2.687(1.054,6.848） | 0.038 |
| CVD | 356(15.2%) |  |  |  |  |
| 5.6≤FPG<6.1mmol/L | 16（8.1%） | 0 | 0 | 0 | 0 |
| FPG<5.6mmol/L | 173（13.6%） | 1.894(1.134,3.163） | 0.015 | 1771(1.021,3.072） | 0.042 |
| 6.1≤FPG<6.9mmol/L | 31（14.8%） | 2.016(1.101,3.692） | 0.023 | 1.805(0.930,3.502） | 0.081 |

Adjusted group: adjusted for age, gender, BMI, hypertension status, 24-h urine volume, albumin, hemoglobin.

Abbreviations: CV-mortality, cardiovascular mortality; CVD, cardiovascular disease; FPG: fasting plasma glucose.
